# The value of health literacy for Global Health and One Health: nurturing the socioecological environment of schools

**DOI:** 10.1093/heapro/daag036

**Published:** 2026-03-11

**Authors:** Orkan Okan, Andrea S Winkler

**Affiliations:** Health Literacy Unit, Department of Health and Sport Sciences, TUM School of Medicine and Health, Technical University of Munich, Munich, Germany; WHO Collaborating Centre for Health Literacy, Department of Health and Sport Sciences, TUM School of Medicine and Health, Technical University of Munich, Munich, Germany; Department of Neurology, TUM University Hospital, and Center for Global Health, TUM School of Medicine and Health, Technical University of Munich, Munich, Germany; Department of Community Medicine and Global Health, Institute of Health and Society, Faculty of Medicine, University of Oslo, Oslo, Norway

**Keywords:** health literacy, One Health, Ottawa Charter, health promotion, schools, settings, health literate schools, health equity

## Abstract

This perspective examines health literacy through a One Health lens, emphasizing its relevance for developing One Health−literate citizens, as advocated by the Lancet One Health Commission's recommendation on school health literacy. It argues for a comprehensive understanding of health literacy across the socioecological continuum, integrating both individual agency and structural influences. Rather than viewing low health literacy as an individual deficiency, this perspective situates it within broader systemic and contextual factors, particularly pertinent to low- and middle-income countries and underserved populations. Building on this premise, the Health-Literate School (HeLit-School) framework offers a whole-of-school approach to advancing health literacy by embedding the concept of organizational health literacy within school systems. Drawing on socioecological and organizational development principles, HeLit-School fosters institutional transformation across all levels of school development, supporting the implementation of holistic, multi-level interventions to enhance health literacy in the school setting. It highlights the critical roles of principals and the importance of adequate resources as structural enablers of sustainable health promotion within educational settings. This perspective calls for integrating agency-based, behavioural approaches with structural, determinant-oriented strategies to strengthen both personal and organizational health literacy in schools. Aligning One Health principles with the HeLit-School framework helps bridge the behavioural and environmental determinants of health. The proposed adaptation of HeLit-Schools for One Health and its contextualization in low- and middle-income countries illustrate how upstream, setting-based interventions can support systemic capacity-building and the formation of health-literate students, educators, and policymakers in relation to One Health.

Contribution to Health Promotion

- Supports development of One Health−literate citizens through socioecological, equity-oriented educational strategies.
- Advances a comprehensive framework linking health literacy with One Health and Global Health perspectives.
- Adapts the Health-Literate School framework for diverse contexts, including low- and middle-income countries, to enhance global applicability.
- Promotes a whole-of-school, organizational health literacy approach to strengthen school-based health promotion.
- Integrates behavioural and structural determinants to create supportive, health-literate school environments.

## Introduction

The recently published report of the ‘Lancet’ One Health Commission links One Health closely to the Global Health agenda (Winkler *et al.* 2025) and, in addition, presents a pathway for a new public health. A key commonality between the two concepts, i.e. One Health and Global Health, is the drawing on traditional health promotion concepts and methodologies, including equity-driven socioecological and upstream determinant-based approaches, collective and collaborative action, systemic transformation, interdisciplinary and intersectoral thinking, and setting-based, whole-of-environment frameworks, to improve population health outcomes. Both concepts also seek to tackle complex health challenges across disciplinary fields and sectors that have become inherent in the 21st century resulting from globalization and the diminishing of disciplinary and sectoral boundaries, as well as the complex, often opaque interaction of socioeconomic, environmental, geographical, political, commercial, technological, digital, and social determinants. While those determinants and their influence on people's lives, including their impact on health and well-being, are well documented (Marmot *et al.* 2012, Donkin *et al.* 2018, World Health Organization 2025), contrary to their impact on animal health and welfare, they may seem intangible or imperceptible in everyday life but hint at the need for system-level structural interventions to address the root causes of ill health and health inequities (Marmot *et al.* 2012, Winkler *et al.* 2025 , World Health Organization 2025). Interestingly, both concepts also recommend utilizing health literacy as a key strategy to achieve their overarching goals. Echoing the World Health Organization’s recommendations to incorporate health literacy into school education (Kickbusch *et al.* 2013, McDaid 2016, World Health Organization 2017, 2021), the Lancet One Health Commission also advocates for One Health literacy in schools, preschools, and tertiary education (Winkler *et al.* 2025 ). Similarly, further Lancet Commissions and Series are emphasizing the adaptation of health literacy strategies in schools (Patton *et al.* 2016, Baird *et al.* 2025) and for improving primary care delivery in low- and middle-income countries (LMICs; Lilford *et al.* 2025). Widespread low levels of health literacy among schoolchildren in many countries underscore the urgent need to invest in school health literacy (Paakkari *et al.* 2020, Stauch *et al.* 2025), especially because higher health literacy levels are linked to improved health and behaviour outcomes (Fleary *et al.* 2018 ) and at school, all school-aged children can be reached regardless of their social background (World Health Organization 2021, Kirchhoff *et al.* 2025). However, most existing population-based studies in child and adolescent populations have been conducted in the European Region or North America, making it difficult to make assumptions about child and adolescent health literacy in LMICs and non-Western countries. Nevertheless, findings across studies reveal that children’s low health literacy levels consistently follow a social gradient, disproportionately affecting children from low socioeconomic family households and reinforcing pre-existing inequalities (Paakkari *et al.* 2019, Kirchhoff *et al.* 2025). Therefore, a global One Health−oriented citizenry, an ambition stemming from the call to enable One Health−literate citizens (Winkler *et al.* 2025), requires a system-wide adaption of early health-promoting school interventions supported by policies dedicating explicit commitment, funding, and social investment. This call has recently been echoed by scholars who emphasize the importance of planetary and One Health literacy in tackling current environmental, health, and social challenges (Jochem *et al.* 2025). One Health literacy is based on the generic concept of health literacy as defined by the European Health Literacy Survey Consortium (Sørensen *et al.* 2012) and describes it as a set of prerequisites ‘to access, understand, appraise and apply all relevant information that are related to One Health in order to make judgments, take decisions and actions in everyday life concerning healthcare, disease prevention and health promotion to sustainably balance the health and quality of life of us humans, the animals and the environment’ (Blankart *et al.* 2024). Public health strategies exclusively focusing on individual-level, behavioural health literacy programmes at school and not accounting for prevalent social and health inequalities are unlikely to achieve sustainable improvements in child and adolescent health literacy across the gradient (Kickbusch *et al.* 2013, Kirchhoff *et al.* 2025). Instead, equity-driven school health literacy requires a structural, setting-based, and policy-level approach as a starting point. However, the systemic-level intervention will on its own not solve the challenges in implementing school health literacy, but it is the quintessential step to foster a climate that enables meaningful action on the ground to improve health literacy, alongside policy support, sustainable funding, improvements in the environment, and professional training.

Given these observations, this viewpoint aims to elucidate the various facets and multiple meanings of health literacy with a focus on One Health and how those can actually support developing One Health−literate citizens, as recommended by the Lancet One Health Commission. In doing so, the focus is on expanding on the recommendations to embrace the full socioecological spectrum provided by health literacy. This perspective will add to Global Health and One Health education frameworks by drawing on health literacy’s inherent attributes of upstream, setting-based, and environmental characteristics, combining behaviour and structure perspectives.

## Understanding health literacy

Health literacy is often referred to as an information management skill enabling people to navigate complex health information ecosystems and healthcare service environments (Nutbeam and Muscat 2021). It represents a rather new tool in the larger public health toolbox of concepts to improve individual and population health outcomes through health-promoting and preventive measures. Conceptually, commonly stressed features of health literacy include information seeking, understanding, critical thinking and appraisal, as well as using information to influence decision-making processes and eventually behaviour (Sørensen *et al.* 2012). Health-literate individuals are also empowered to comprehend public health topics, concepts, and strategies and discern health facts from health mis- and disinformation, which has become more important since the COVID-19 pandemic and the associated infodemic (Okan *et al.* 2020a, Paakkari and Okan 2020). Health literacy draws upon knowledge and competencies, yet it is a separate construct. Like most other educational, pedagogical, or psychological constructs, health literacy integrates knowledge and competencies into its own fabric and even presupposes them (Nutbeam and Muscat 2021, Sørensen *et al.* 2021), but it is oriented towards and shaped by its objective to enable individuals, especially to make informed health choices rather than just possess factual health knowledge without being able to use information.

However, this conceptualization falls short of the true depth of health literacy that could contribute to achieving the goals set out in the Global Health and One Health agendas (Winkler *et al.* 2025). Aside from understanding health literacy as a prerequisite set of health skills (Nutbeam and Muscat 2021), health literacy has been conceptualized as a relational model shifting from viewing low health literacy as a behavioural deficit to account for the impact of systems, environmental conditions, and upstream determinants (Parker and Ratzan 2010, Brach *et al.* 2012, Sørensen *et al.* 2021, Kirchhoff *et al.* 2025). Health literacy reflects both behavioural and structural aspects, which can be beneficial, especially for LMICs and further underserved communities, as the individual is viewed in the light of the interactions with systems and available resources. Setting the relational model as a conceptual backbone, health literacy’s full scope includes two interdependent facets: (i) agency and behaviour change models linked to knowledge and competency approaches addressing individual-level factors (e.g. personal health literacy) and (ii) structure and social change models linked to socioecological and setting approaches addressing environmental-level factors (e.g. systemic or organizational health literacy; Brach *et al.* 2012, Abel and Benkert 2022, Kirchhoff *et al.* 2025).

## The agency dimension of health literacy

On the agency side of the equation, an increased understanding of health information and the knowledge of how to utilize information is meant to improve people’s health and well-being outcomes (Sørensen *et al.* 2012, Nutbeam and Muscat 2021, Abel and Benkert 2022, Levin-Zamir *et al.* 2025). Health-literate individuals are better equipped to access and navigate healthcare and information systems, understand health messages and see through false health claims, reflect on treatment options and medication instructions, communicate confidently about health, and frequently engage in health-promoting and preventive measures (Parker and Ratzan 2010, Sørensen *et al.* 2012, Paakkari and Okan 2020). Improved personal health literacy may also mediate between unequally distributed socioeconomic factors and personal resources in disadvantaged and underserved populations, contributing to reducing health disparities and promoting health equity (Paakkari *et al.* 2019, Stormacq *et al.* 2019). The Lancet One Health Commission suggests that health literacy presupposes media literacy (Winkler *et al.* 2025) and both concepts indeed represent conceptual synergies, even overlapping in some of their dimensions (Schulenkorf *et al.* 2021, World Health Organization 2021). A most welcome side effect is that a media or digital literacy curriculum at school can be used to teach health literacy (Schulenkorf *et al.* 2021, Stauch *et al.* 2025), while avoiding putting additional burden on schools and their already overcrowded curriculum, low resources, and often understaffed workforce (Schulenkorf *et al.* 2021, Kirchhoff *et al.* 2025). If linked to health-related topics, media or digital literacy education at school can reinforce children’s abilities to, e.g. distinguish correct information and facts from health misinformation, critically assess health claims, and recognize underlying, often hidden commercial interests (Stauch *et al.* 2025). This will empower children and adolescents in their own right and enable them to act more autonomously, independently, and self-determinedly, capable of managing uncertainty and fear more effectively and reducing their susceptibility to propagandist and populist messages—as called for by the Lancet One Health Commission (Winkler *et al.* 2025).

## The structural dimension of health literacy

The structural side of health literacy mirrors the agency aspect by focusing on how health systems and policy environments can make it easier for individuals—in particular those with low resources—to act on health information and receive the services they need by serving the user demand perspective. This paradigm of health literacy is linked to health equity thinking and suggests planning and implementing structural and upstream health-promoting and preventive interventions to create health literacy−friendly, −supportive, and −responsive environments (Parker and Ratzan 2010, Kickbusch *et al.* 2013, Kirchhoff *et al.* 2025). Effective policy and system-level interventions can ensure the creation of user-centric settings and structures to enable people to navigate environments (healthcare and information systems) more easily, access care services, choose between care options, and engage in overall healthy behaviours so that these actions are not solely dependent on the individual's skill, behaviour, or responsibility (Parker and Ratzan 2010, Kickbusch *et al.* 2013, Sørensen *et al.* 2021). This may include addressing several avenues for improvement, among which are making health information more accessible and actionable through clear, culturally appropriate health communication programmes and redesigning healthcare and information systems to reduce complexity and support informed decision-making (Brach *et al.* 2012, Sørensen *et al.* 2021). The exact design will depend on the cultural and geographical context and might also be fundamentally different between LMICs and high-income countries (HICs). Further strategies to tackle the structural dimension of health literacy include harnessing the potential that lies in developing health-literate organizations and programmes to train highly skilled workforces in (public) health and education (Brach *et al.* 2012, Kickbusch *et al.* 2013, Sørensen *et al.* 2021, Kirchhoff *et al.* 2025). This approach has been included in the WHO definition of health literacy, highlighting that health literacy is ‘mediated by the organisational structures and availability of resources’ (Nutbeam and Muscat 2021). In addition, sustaining needs-based and equity-driven health literacy programmes will also require engaging the public, private, and community sectors in coordinated policy developments and multi-sectoral partnerships (Kickbusch *et al.* 2013 , Sørensen *et al.* 2021), also reflected well by the Lancet One Health Commission (Winkler *et al.* 2025). This strategy draws on socioecological and health promotion assumptions (e.g. settings, contexts, environments, and ‘Health in All Policies’), which are connected to ensuring that health literacy capacities are developed and supported across different sectors of society (Kickbusch *et al.* 2013) and in relation to planetary and one health (Jochem *et al.* 2025).

## A socioecological lens to school health literacy in the context of the One Health agenda

While the agency approach to health literacy may support the One Health education framework to teach children One Health knowledge and skills (Winkler *et al.* 2025), it will need the structural health literacy framework to effectively contribute to the socioecological health promotion thinking and institutionalization of society-wide implementation as endorsed by the Lancet One Health Commission. Transformative systemic change and sustained efforts in education will require a whole-of-school implementation plan, informed by key organizational development theories and concepts, which have guided the development of the Health-Literate School (HeLit-School) framework. Incorporating the organizational health literacy model (Brach *et al.* 2012) and synergetic to the WHO Health Promoting School (HPS) framework (World Health Organization and UNESCO 2021), HeLit-School represents the setting approach to promote health literacy and create a supportive school environment. HeLit-School provides eight interconnected standards, each including six measurable indicators, to support the school setting to develop into a health-literate school (Kirchhoff *et al.* 2025). HeLit-School addresses four target points in the socioecological environment of the school, including schoolchildren, teachers and staff, school principals, and the wider school environment. The framework can be utilized to strategically guide comprehensive One Health literacy school interventions (Table 1), while the HeLit-School standards and indicators also reflect the five levels of influence of the socioecological model (Golden and Earp 2012). For the implementation of socioecological school interventions, the school principal is a key influential authority to enable health activities in the school setting. Principals with higher levels of health literacy implement school health promotion programmes more frequently (Dadaczynski *et al.* 2020, Betschart *et al.* 2022, Meyer *et al.* 2025), positioning them as a key intervention target at the structural level, where system-level influences on child/adolescent health outcomes and health literacy are addressed indirectly. However, the available resources, more precisely, financial, personnel, and time resources, represent a primary success factor for any school health activity (Kirchhoff *et al.* 2025), which is why school health literacy programmes must be underpinned by binding legislative and fiscal policies to be effective in the long term.

**Table 1:** Standards of a HeLit-School and their integration with One Health objectives.

HeLit-School standards (Kirchhoff *et al.* 2025)	HeLit-School adaption to One Health
Standard 1: Include health literacy in the school’s mission statement	Evolve the mission statement to explicitly incorporate One Health literacy, emphasizing education about the links between human health, animal health, and the health of the wider ecosystem.
Standard 2: Health literacy as part of school development	Integrate One Health principles into school policies and development plans, fostering curriculum development that reflects the interdependence of health across species and environments.
Standard 3: Promote and enhance health literacy in daily school life	Include activities and practices in everyday school routines and projects that raise awareness for One Health topics, such as zoonotic diseases, environmental sustainability, and ecosystem and planetary health.
Standard 4: Health literacy of students	Promote schoolchildren’s One Health literacy, encompassing not only personal and community health and well-being but also understanding of how animal and environmental health impacts human health and well-being, encouraging systems thinking and transdisciplinary learning.
Standard 5: Health-literate school staff	Train teachers and staff, including school principals, on One Health concepts so they can confidently incorporate these themes into teaching and engage schoolchildren in interconnected health topics along the One Health spectrum.
Standard 6: Health-literate communication at school	Implement communication that provides precise messages and easy-to-understand information about human, animal, and environmental health issues, fostering a One Health dialogue within and beyond school.
Standard 7: Enhance health literacy in the school environment	Create a school environment that includes One Health principles, e.g. supporting biodiversity on school grounds, promoting ecological awareness, and minimizing environmental hazards and health risks, and also encouraging out-of-classroom teaching.
Standard 8: Networking and cooperation	Build partnerships involving health professionals, veterinarians, environmental scientists, and the community to support school health initiatives that reflect the One Health approach.

The left column displays the titles of the original standards of the HeLit-School framework, and the right column shows the descriptors for each standard to match One Health concepts. Each descriptor of the original framework was reviewed in the One Health context, but it still must be carefully adapted to reflect local contexts, especially in LMICs. The original standards can be accessed in the publication by Kirchhoff *et al*. (2025).

**Figure 1:**
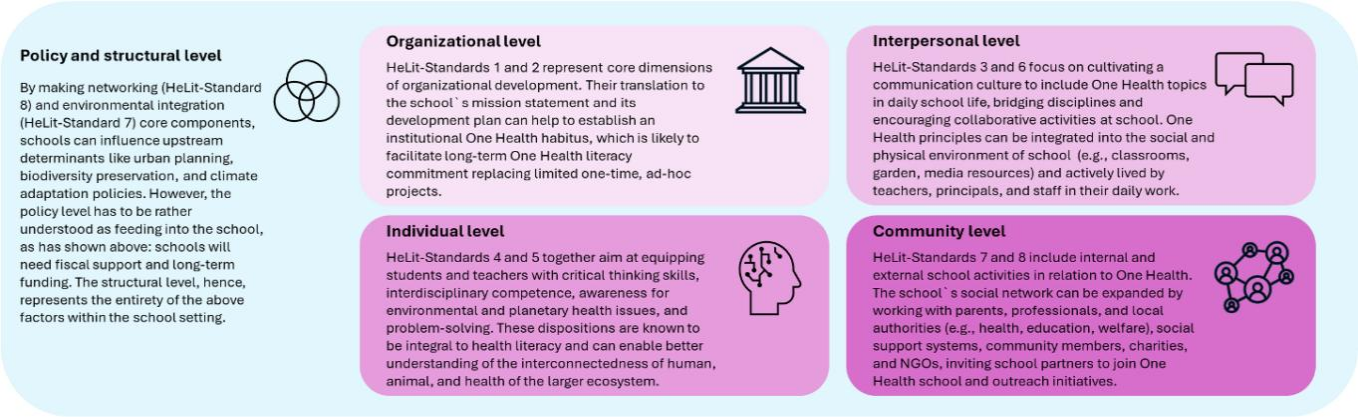
One Health integrated into the HeLit-school framework and embedded in the socioecological model. The four squares depict four levels of influence as suggested by the socioecological model (individual, interpersonal, community, and organizational). Those influences occur and are located directly at the school setting. They are embedded in the wider societal structures where policies and legislations (the fifth level of influence) shape funding, investment, support, and commitment to education. The policy and structural level of influence shapes whether activities can be implemented at school, meaning that the other four levels of influence are highly dependent on the directions and strategies set out at the policy and structural level.

This integrated expansion of the HeLit-School framework is aligned with the One Health principles but expands the suggested education framework of the Lancet One Health Commission to support a holistic, socioecological school health literacy framework, encouraging schoolchildren, teachers, staff, principals, parents, and educational administrators and policymakers to understand and act on the interconnected determinants of One Health in their communities and beyond. Standards 4 and 5 of the HeLit-School framework already address several aspects of the Commission’s Recommendation 9 to achieve a One Health−literate global citizenry (Winkler *et al.* 2025). In the report, these standards are especially reflected by One Health literacy ‘[…] must be mainstreamed within all levels of preschooling and primary and secondary schooling’, ‘One Health literacy implies building capabilities and capacity among the younger generation’ (Recommendations 9B and 9C, p. 51), and ‘One Health literacy entails professional lifelong learning and development’ (Recommendation 9D, p. 51; Winkler *et al.* 2025). The remaining HeLit-School standards go beyond these elements and introduce multiple new dimensions across the socioecological spectrum by which the One Health goals, e.g. socioecological transformation, health-promoting synergies, and social and environmental determinants of health, could be supported more effectively. When situating the HeLit-School framework and One Health within the socioecological model, they can be merged and examined across the five levels of socioecological influence: individual, interpersonal, organizational/institutional, community, and policy/structural levels (Fig. 1; Golden and Earp 2012).

While the HeLit-School framework was developed especially with German schools in mind, and in collaboration with German and Austrian research, practice, and policy experts from education, public health, and medicine, the integral framework indicators (Kirchhoff *et al.* 2025) might need to be adapted to meet needs, norms, requirements, standards, and languages available in LMICs (Table 2). The implementation of the HeLit-School framework has to consider several aspects that may support the success of the programme (World Health Organization 2021), including whole-school anchoring of health literacy, policy support, committed school leadership, cross-sector networks, alignment with existing HPS structures (Dadaczynski *et al.* 2020, Schulenkorf *et al.* 2021, Meyer *et al.* 2025 ), and the use of self-assessment instruments such as the Organisational Health Literacy of Schools Questionnaire (OHLS-Q) (Kirchhoff *et al.* 2025). At the same time, typical challenges, including competing curricular priorities, limited resources, the need for sustained leadership and professional development, lack of time, fragmented responsibilities, and insufficient monitoring mechanisms (Okan *et al.* 2020b, World Health Organization 2021), should be carefully considered before transferring and adapting the framework to LMIC contexts. Especially, teaching content for health literacy learning materials will need to be carefully revised, modified, and also adapted to culturally fit the LMIC context. Nevertheless, the general multi-level standard-based approach of the HeLit-School framework may remain and serve as a structural foundation to deliver a whole-of-school intervention rather than an individual-level intervention.

**Table 2:** One Health recommendations (Winkler *et al*. 2025), HeLit-School standards (Kirchhoff *et al*. 2025), socioecological levels (Golden and Earp 2012), and adaptation to LMIC context.

Recommendations to achieve a One Health−literate global citizenry (Winkler *et al.* 2025)		HeLit-School standards (Kirchhoff *et al.* 2025)	Socioecological levels (Golden and Earp 2012)	Suggested adaptations for LMICs
Goals | prerequisites on policy and governance levels	Goals | actionable interventions at school level			
1. Advance One Health operationalization, implementation, and institutionalization through public−private partnerships	–	Std 2: School development Std 8: Networking and cooperation	Organizational Policy/structural Community	Context specificity: adapt school development plans to, e.g. meet local cultural requirements, social norms and roles, community needs, and economic circumstances (also relevant for goal #2 of actionable interventions) Collaborate with local leaders and elders, informal networks, local (traditional) health authorities, and low-cost partnerships with non-governmental organizations, farmers, agencies, community groups, and faith-based organizations (also relevant for goal #2 of actionable interventions)
	2. Mainstream One Health knowledge and principles at all levels of preschool, primary, and secondary schooling	Std 1: Mission statement Std 2: School development Std 6: Communication Std 7: School environment Std 8: Networking and cooperation	Organizational Policy/structural Community	Adapt programmes and communication to local health promotion and prevention topics, local assessment needs, and infrastructure Consider differences between rural and urban area schools and schools in low-resource districts Empower communities to tell their own ‘health stories’ and drive community-led advocacy projects Integrate indigenous knowledge, take into account wider family environment Translate into informal and non-formal education (radio, storytelling, community workshops)
	3. Build capabilities and capacity among youth (leadership, communication, negotiation, teamwork, innovative problem-solving)	Std 3: Daily school life Std 4: Health literacy of students	Individual Interpersonal	Prioritize local health and well-being topics, combine with local health knowledge, adapt materials to meet local context and norms, and acknowledge interests and attitudes of children Use locally relevant, low-tech solutions (e.g. learning and teaching modes, school gardening, agriculture and sustainable farming, water harvesting, waste management, and hygiene) If necessary, adapt to implementing distant- and peer-learning techniques Run student-led advocacy and environmental projects, involve local councils, and integrate indigenous knowledge, experience, and attitudes
	4. Facilitate professional lifelong learning and development	Std 5: Health-literate staff	Organizational Policy/structural	Hands-on teaching strategies, workforce development, communication techniques Evaluate curriculum for in-service and pre-service teachers and adapt training materials to local (health, social, and cultural) context Use peer-to-peer teacher training, mobile learning platforms, and adapt/blend technology to context (e.g. low-bandwidth digital resources) Flexibility to account for low resources, balancing quality with scale regarding cost-effectiveness Mentorship for more effective practical experience
5. Build One Health literacy across all levels of the political spectrum for governance transformation		Relevant across Std 1–8	Organizational Policy/structural Community	Advocate for policies and funding across the political spectrum and for school health promotion, use tailored messages (also relevant for goal #1 of actionable interventions) Form alliances that include all relevant actors, empower communities, enable meaningful participation and ownership, and engage the media and civil society (also relevant for goal #1 of actionable interventions) Translate evidence from successful case studies benefitting long-term population health goals, provide reasons for legitimacy, highlight the return-on-investment effects, seek low-cost solutions if necessary and consider local realities, including traditional knowledge (also relevant for goal #1 of actionable interventions) Use grassroots movements and bottom-up and top-down strategies (also relevant for goal #1 of actionable interventions)

The first column entails the five original goals of Recommendation 9 of the Lancet One Health Commission to achieve a One Health−literate global citizenry (Winkler *et al.* 2025), separated into two categories of (i) prerequisites and (ii) actionable interventions; the second column refers to the HeLit-School standards (Kirchhoff *et al.* 2025) linked to these two categories; the third column introduces the socioecological levels (Golden and Earp 2012); the last column presents an *ad hoc* adaptation to LMICs and their societal and cultural context. To avoid duplicate mentions, LMIC adaptations that apply to more than one One Health goal are highlighted in the cells.

## Conclusion

The HeLit-School framework becomes an incubator to promote aspects of One Health as a whole-of-school approach, aiming at influencing the next generation to become One Health advocates and champions. However, in order to achieve this goal, this viewpoint has shown that schools themselves need to be ‘influenced’ on their structural levels, which can only be achieved if governments value them and their mandate for education by guaranteeing continuous funding and including health and education in all policies. Investing in education is a core requirement for any sustainable strategy to promote Global Health and One Health. While the former focuses on improving health equity and addressing widespread health challenges worldwide, the latter recognizes the interconnectedness of human, animal, plant, and ecosystem health and is firmly based on the One Health ethics as described in the Introduction. Health literacy connects both concepts by its integral socioecological lens to address these problems in a highly information-based and -saturated 21st century world. Health literacy strategies in general, and especially when implemented early in schools in both LMICs and HICs, can contribute to achieving the goals of Global Health and One Health. However, this will require shifting from a behavioural to a systemic perspective, which organically includes human behaviour, and moving beyond the sole individual focus towards adopting a socioecological perspective, in particular at school. While individuals may have the responsibility for engaging with their health and well-being, only systems and policymakers can establish enabling and supportive environments and sustainable health and education policies. The flourishing of health literacy within the socioecological environment not only enables its full potential and maximizes its impact but also represents a clear added value if adapted systematically to Global Health and One Health strategies. Ahead of the 40th anniversary of the Ottawa Charter for Health Promotion in 2026, it is worth to recite the then Director General of the WHO, Dr Halfdan Mahler, who urged to ‘recognize health and its maintenance as a major social investment that is part of the overall process of social learning and support health literacy as a major aspect of personal learning and development’ (Mahler 1986, p. 411). The report of the Lancet One Health Commission represents a paradigm shift through the adoption of key health promotion principles, grounded in interdisciplinary and multi-sectoral, (socio)ecological, and equity-driven frameworks. Taking into account the convergence of human, animal, and environmental factors, the Ottawa Charter's mandate to implement a ‘new public health’ is being revived. Long-term investment in health literacy will support the agendas of Global Health and One Health and contribute to tackling challenges across the health spectrum in this age of new public health.

## Data Availability

No primary data was used for this perspective.

## References

[daag036-B1] Abel T, Benkert R. Critical health literacy: reflection and action for health. Health Promot Int 2022;37:2022. 10.1093/heapro/daac114 PMC9434639 36047637

[daag036-B2] Baird S, Choonara S, Azzopardi PS et al A call to action: the second Lancet Commission on adolescent health and wellbeing. Lancet 2025;405:1945–2022. 10.1016/S0140-6736(25)00503-3 40409329

[daag036-B3] Betschart S, Sandmeier A, Skedsmo G et al The importance of school leaders’ attitudes and health literacy to the implementation of a health-promoting schools approach. Int J Environ Res Public Health 2022;19:14829. 10.3390/ijerph192214829 36429547 PMC9690102

[daag036-B4] Blankart CR, Gani SM, de Crimlisk H et al Health literacy, governance and systems leadership contribute to the implementation of the One Health approach: a virtuous circle. Health Policy 2024;143:105042. 10.1016/j.healthpol.2024.105042 38518391

[daag036-B5] Brach C, Keller D, Hernandez LM et al Ten attributes of health literate health care organizations. 2012. https://nam.edu/perspectives-2012-ten-attributes-of-health-literate-health-care-organizations/ (7 August 2025, date last accessed).

[daag036-B6] Dadaczynski K, Rathmann K, Hering T et al The role of school leaders’ health literacy for the implementation of health promoting schools. Int J Environ Res Public Health 2020;17:1855. 10.3390/ijerph17061855 32178457 PMC7142764

[daag036-B7] Donkin A, Goldblatt P, Allen J et al Global action on the social determinants of health. BMJ Glob Health 2018;3:e000603. 10.1136/bmjgh-2017-000603 PMC5759713 29379648

[daag036-B8] Fleary SA, Joseph P, Pappagianopoulos JE. Adolescent health literacy and health behaviors: a systematic review. J Adolesc 2018;62:116–27. 10.1016/j.adolescence.2017.11.010 29179126

[daag036-B9] wGolden SD, Earp JAL. Social ecological approaches to individuals and their contexts: twenty years of health education & behavior health promotion interventions. Health Educ Behav 2012;39:364–72. 10.1177/1090198111418634 22267868

[daag036-B10] Jochem C, Doyle G, Sørensen K et al A call for a shared future vision for Planetary and One Health Literacy. Health Promot Int 2025;40:2025. 10.1093/heapro/daaf200 PMC12648243 41293977

[daag036-B11] Kickbusch I, Pelikan JM, Apfel F et al Health literacy: the solid facts. 2013. https://iris.who.int/handle/10665/326432 (7 August 2025, date last accessed).

[daag036-B12] Kirchhoff S, Krudewig C, Okan O. Organizational health literacy of schools in Germany: results of a cross-sectional study. Health Promot Int 2025;40:2025. 10.1093/heapro/daaf112 PMC12318714 40754692

[daag036-B13] Levin-Zamir D, van den Broucke S, Bíró É et al Measuring digital health literacy and its associations with determinants and health outcomes in 13 countries. Front Public Health 2025;13:1472706. 10.3389/fpubh.2025.1472706 40182520 PMC11966570

[daag036-B14] Lilford RJ, Daniels B, McPake B et al Policy and service delivery proposals to improve primary care services in low-income and middle-income country cities. Lancet Glob Health 2025;13:e954–66. 10.1016/S2214-109X(24)00536-9 40288403

[daag036-B15] Mahler H . Address: Dr Halfdan Mahler. Health Promot Int 1986;1:409–11. 10.1093/heapro/1.4.409 28180252

[daag036-B16] Marmot M, Allen J, Bell R et al WHO European review of social determinants of health and the health divide. Lancet 2012;380:1011–29. 10.1016/S0140-6736(12)61228-8 22964159

[daag036-B17] McDaid D . Investing in Health Literacy: What do We Know About the Co-benefits to the Education Sector of Actions Targeted at Children and Young People? Copenhagen: World Health Organization, Regional Office for Europe, European Observatory on Health Systems, 2016. https://iris.who.int/handle/10665/331987 29144632

[daag036-B18] Meyer M, Dadaczynski K, Messer M et al Association of school leaders’ COVID-19 health literacy with the implementation of health promotion in schools in Germany: a quantitative cross-sectional study. BMC Public Health 2025;25:2875. 10.1186/s12889-025-24196-9 PMC12369071 40841892

[daag036-B19] Nutbeam D, Muscat DM. Health promotion glossary 2021. Health Promot Int 2021;36:1578–98. 10.1093/heapro/daaa157 33822939

[daag036-B20] Okan O, Bollweg TM, Berens E-M et al Coronavirus-related health literacy: a cross-sectional study in adults during the COVID-19 infodemic in Germany. Int J Environ Res Public Health 2020a;17:5503. 10.3390/ijerph17155503 32751484 PMC7432052

[daag036-B21] Okan O, Paakkari L, Dadaczynski K. Health literacy in schools: State of the art. Schools for Health in Europe Network Foundation. Funded by the European Commission. Haderslev, Denmark. 2020b. https://www.schoolsforhealth.org/sites/default/files/editor/fact-sheets/factsheet-2020-english.pdf (12 February 2026, date last accessed).

[daag036-B22] Paakkari L, Okan O. COVID-19: health literacy is an underestimated problem. Lancet Public Health 2020;5:e249–50. 10.1016/S2468-2667(20)30086-4 32302535 PMC7156243

[daag036-B23] Paakkari L, Torppa M, Mazur J et al A comparative study on adolescents’ health literacy in Europe: findings from the HBSC study. Int J Environ Res Public Health 2020;17:3543. 10.3390/ijerph17103543 32438595 PMC7277198

[daag036-B24] Paakkari LT, Torppa MP, Paakkari O-P et al Does health literacy explain the link between structural stratifiers and adolescent health? Eur J Public Health 2019;29:919–24. 10.1093/eurpub/ckz011 30753409

[daag036-B25] Parker R, Ratzan SC. Health literacy: a second decade of distinction for Americans. J Health Commun 2010;15:20–33. 10.1080/10810730.2010.501094 20845190

[daag036-B26] Patton GC, Sawyer SM, Santelli JS et al Our future: a Lancet commission on adolescent health and wellbeing. Lancet 2016;387:2423–78. 10.1016/S0140-6736(16)00579-1 27174304 PMC5832967

[daag036-B27] Schulenkorf T, Krah V, Dadaczynski K et al Addressing health literacy in schools in Germany: concept analysis of the mandatory digital and media literacy school curriculum. Front Public Health 2021;9:687389. 10.3389/fpubh.2021.687389 34291029 PMC8287418

[daag036-B28] Sørensen K, Levin-Zamir D, Duong TV et al Building health literacy system capacity: a framework for health literate systems. Health Promot Int 2021;36:i13–23. 10.1093/heapro/daab153 34897445 PMC8672927

[daag036-B29] Sørensen K, van den Broucke S, Fullam J et al Health literacy and public health: a systematic review and integration of definitions and models. BMC Public Health 2012;12:80. 10.1186/1471-2458-12-80 22276600 PMC3292515

[daag036-B30] Stauch L, Renninger D, Rangnow P et al Digital health literacy of children and adolescents and its association with sociodemographic factors: representative study findings from Germany. J Med Internet Res 2025;27:e69170. 10.2196/69170 40324766 PMC12089873

[daag036-B31] Stormacq C, van den Broucke S, Wosinski J. Does health literacy mediate the relationship between socioeconomic status and health disparities? Integrative review. Health Promot Int 2019;34:e1–17. 10.1093/heapro/day062 30107564

[daag036-B32] Winkler AS, Brux CM, Carabin H et al The Lancet One Health Commission: harnessing our interconnectedness for equitable, sustainable, and healthy socioecological systems. Lancet 2025;406:501–70. 10.1016/S0140-6736(25)00627-0 40683291

[daag036-B33] World Health Organization . World report on social determinants of health equity. 2025. https://iris.who.int/handle/10665/381152 (9 August 2025, date last accessed).

[daag036-B34] World Health Organization . Shanghai declaration on promoting health in the 2030 Agenda for Sustainable Development. Health Promot Int 2017;32:7–8. 10.1093/heapro/daw103 28180270

[daag036-B35] World Health Organization . Health Literacy in the Context of Health, Well-Being and Learning Outcomes- the Case of Children and Adolescents in Schools: Concept Paper. Copenhagen: WHO Regional Office for Europe. 2021. https://iris.who.int/handle/10665/344901 (7 August 2025, date last accessed).

[daag036-B36] World Health Organization and UNESCO . Making every school a health-promoting school: Implementation guidance. 2021. https://iris.who.int/handle/10665/341908 (7 August 2025, date last accessed).

